# Case report: successful lipid resuscitation in multi-drug overdose with predominant tricyclic antidepressant toxidrome

**DOI:** 10.1186/1865-1380-5-8

**Published:** 2012-02-02

**Authors:** Martyn Harvey, Grant Cave

**Affiliations:** 1 Emergency Medicine Research, Waikato Hospital, Pembroke Street, Hamilton, New Zealand; 2 Hutt Hospital, High Street, Lower Hutt, New Zealand

## Abstract

We report a case of profound neurologic and cardiovascular manifestations of tricyclic antidepressant intoxication following self-poisoning with multiple pharmaceuticals including amitriptyline in excess of 43 mg/kg, in a 51-year-old male. Institution of mechanical ventilation, volume expansion, systemic alkalinisation (pH 7.51), and intermittent bolus metaraminol resulted in QRS narrowing but failed to resolve the developed shock. One 100-ml bolus of 20% lipid emulsion followed by a further 400 ml over 30 min was administered with restoration of haemodynamic stability, thereby curtailing the need for ongoing vasopressor medications. Assayed blood levels were consistent with the 'lipid sink' being a major effecter in the observed improvement.

## Background

Therapeutic use of intravenous lipid emulsion (ILE) in the arrested patient secondary to lipophilic cardiotoxin overdose is increasingly reported, with numerous documented cases of successful resuscitation outcome [1,2]. Clinical experience with lipid rescue resuscitation, coupled with a dearth of reported adverse sequelae attributable to ILE administration, has more recently seen use of lipid emulsions extend beyond that of overt cardiac arrest to instances of lesser degrees of lipophilic-toxin-induced haemodynamic instability.

Few data exist, however, to guide the physician contemplating ILE use in the deteriorating patient when multiple therapeutic options remain yet untried. Specifically, the role of ILE in hemodynamic instability secondary to tricyclic antidepressant (TCA) overdose has been the subject of few pre-clinical studies [3,4]. We report a case of multi-drug overdose with predominant TCA toxicity that exhibited ongoing hypotension after systemic alkalinisation, yet before infusion of vasopressor medications, which responded to ILE loading.

## Case presentation

**Figure 1:**
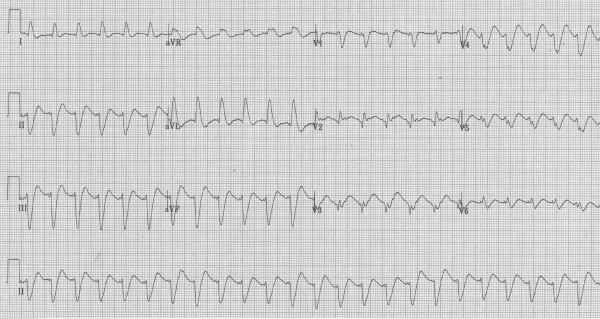
**Twelve-lead electrocardiogram at presentation (time 60 min)**.

A 51-year-old 75-kg man with a background history of ischaemic heart disease, chronic back pain, and depression ingested amitriptyline in excess of 43 mg/kg (> 65 × 50-mg tablets) and unknown quantities of quetiapine, citalopram, metoprolol, quinapril, and aspirin in a deliberate act of self-poisoning. At ambulance arrival (time approximately 40 min after ingestion) he was agitated and poorly co-operative, with a heart rate of 160 bpm and blood pressure 100/70. En route to hospital he became unresponsive and then suffered a generalised seizure, which was terminated with 4 mg intravenous midazolam. On arrival to our tertiary care facility (time 60 min following ingestion), the Glasgow Coma Scale (GCS) score was three, temperature was 37.6°C, pupils were dilated (4 mm), heart rate was 150 beats per minute, blood pressure was 112/82 mmHg, and serum glucose 14.0 mmoll^-1^. A 12-lead electrocardiogram (ECG; Figure 1) revealed a wide complex tachycardia with QRS duration of 180 ms and a prominent R wave in aVR, supporting a clinical diagnosis of tricyclic antidepressant cardiotoxicity.

One-litre 0.9% saline and 50 ml 8.4% sodium bicarbonate were administered intravenously. He subsequently underwent endotracheal intubation following administration of midazolam 5 mg and suxamethonium 100 mg. Mechanical ventilation was initiated and titrated to an end-tidal CO_2 _of 30 mmHg. A gastric tube was placed and 50 g activated charcoal instilled. A further 1-l 0.9% saline was administered intravenously and an additional 300 ml 8.4% sodium bicarbonate injected in divided aliquots (50 ml) to an arterial pH of 7.51 (serum bicarbonate 35.6 mmol/l, sodium 141 mmol/l, potassium 3.4 mmol/l).

**Figure 2:**
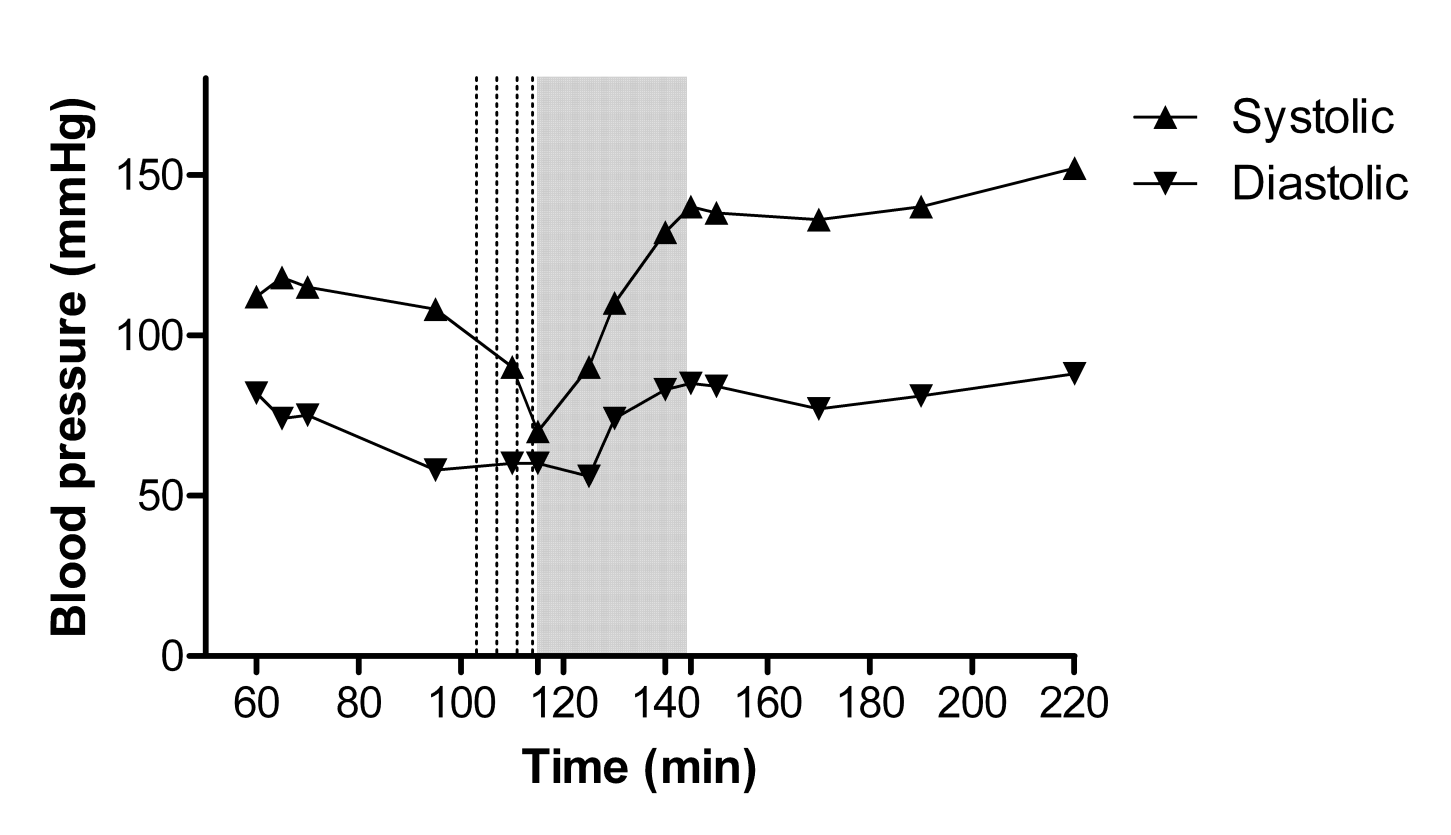
**Blood pressure vs. time from drug ingestion**. *Dotted lines *represent metaraminol injection. *Grey panel *represents duration of ILE infusion.

ECG QRS duration narrowed to 96 ms. However, despite 8 mg metaraminol delivered in 2-mg increments, the blood pressure deteriorated to 70/58 mmHg (pulse rate 130 beats per minute; Figure 2) at time 115 min. Given the ongoing haemodynamic instability, a decision was made to undertake lipid rescue treatment while preparations were made for central line insertion and anticipated vasopressor infusion.

At 115 min after drug ingestion, 100 ml 20% lipid emulsion (Intralipid^®^, Fresenius Kabi) was injected over 1 min followed by a further 400 ml over 30 min. Following administration ECG QRS duration narrowed further to 80 ms, the heart rate was 120 beats per minute, and BP 140/80 mmHg. Serial ECG parameters (QRS duration, QTc) to 205 min are presented in Table 1. Thereafter the patient remained haemodynamically stable. He required no further inotropic/vasoactive medications at any point during his Emergency Department or ICU admission.

**Table 1:** Electrocardiogram QRS duration and QTc according to time from ingestion.

Time after ingestion (min)	60	99	113	145	205
QRS duration (ms)	180	107	96	80	82
QTc	0.57	0.54	0.53	0.51	0.50

ILE infusion time 115-140 min.

Blood was drawn immediately prior to ILE administration, and at 5, 15, 35, and 90 min post ILE commencement (corresponding to 110, 115, 125, 145, and 205 min after initial ingestion) for later determination of plasma amitriptyline and triglyceride concentration. All samples were centrifuged at 3 000 g for 10 min effecting partial visual separation of more lipaemic plasma above from more aqueous plasma below. Blood was then frozen in an upright position to -15°C before undergoing manual cleavage of separated plasma from the buffy coat and red cell mass. The upper 50% of centrifuged plasma (nominally *top*) was then separated from the lower 50% (nominally *bottom*) in an attempt to obtain contemporaneous samples exhibiting a gradient of lipaemia. All samples then underwent assay for plasma amitriptyline by high-performance gas chromatography with the mass selection method and plasma triglyceride estimation by a commercial laboratory. Plasma amitriptyline and triglyceride concentrations are presented in Table 2.

**Table 2:** Plasma amitriptyline and triglyceride levels according to time from ingestion.

Time after ingestion (min)	110	115	125	145	205
Amitriptyline top (nmol/l)	4,100	4,620	4,960	14,440	4,930
Triglyceride top (mmol/l)	1.2	5.1	11.4	35.9	19.6
Amitriptyline bottom (nmol/l)	4,290	4,910	5,640	9,580	4,680
Triglyceride bottom (mmol/l)	1.4	4.8	6.8	9.6	12.5

ILE infusion time 115-140 min.

Triglyceride normal range 0.3-1.9 mmol/l

The patient was subsequently transferred to the ICU with ongoing bicarbonate infusion. Serum lipase was 18 U/l (normal range 13-60 U/l) 24 h after lipid infusion. Electrocardiogram QRS duration was noted to have normalised completely on day 2. Extubation occurred on day 3 with ICU discharge on the same day following development of aspiration pneumonia requiring antibiotic therapy. He was discharged neurologically intact to the psychiatry service on day 7.

## Conclusions

We report a case of self-poisoning with multiple pharmaceuticals wherein the dose of amitriptyline taken, initial clinical course, and amitriptyline levels prior to the use of ILE suggest a high potential for lethality [5]. In this case there was a rapid and marked haemodynamic improvement following ILE infusion, curtailing the anticipated need for further vasopressor medications. Severe amitriptyline toxicity may result in central nervous system depression, seizures, hypotension, and abnormalities to cardiac conduction characterised by electrocardiogram QT and QRS prolongation, in addition to supraventricular and ventricular arrhythmias. Sodium bicarbonate is viewed as specific antidotal therapy in TCA-induced cardiotoxicity [6]. Standard management of severe poisoning entails aggressive supportive care including mechanical ventilation and vasopressor infusion for hypotension refractory to both volume expansion and sodium bicarbonate infusion.

Toxicologic analysis in the present case strongly supports a pharmacokinetic mechanism as one effector in the observed improvements in cardiovascular performance. ILE infusion was intimately associated with both resolution of shock and further reduction in QTc and ECG QRS duration, suggesting amelioration of amitriptyline-induced cardiotoxicity. An increase in total plasma amitriptyline concentration was observed to correlate with triglyceride elevation following ILE infusion. This suggests substantial intravascular lipid sequestration of lipophilic amitriptyline (logP 5.0 [5]), consistent with the "lipid sink" hypothesis first proposed by Weinberg in 1998 [7], with greater amitriptyline levels being seen in the more lipaemic ('top') samples than in those of the less lipaemic 'bottom'. Free amitriptyline levels, while unmeasured in the present case, are likely to have fallen in line with the work of French et al. who reported a 47% predicted lipid extraction efficiency with ILE application in vitro [8]. Persisting haemodynamic stability despite a decline in both triglyceride and amitriptyline levels at 205 min, most notably in the more lipaemic ('top') samples, furthermore suggests a role for circulating lipid in augmentation of toxin redistribution. Greater blood carriage of amitriptyline afforded by lipid infusion potentially serving to speed drug transport to biologically inert sites.

Limitations in these data nevertheless preclude a definitive causal linkage between assayed amitriptyline elevation and induced hypertriglyceridaemia. Amitriptyline levels may have increased regardless of ILE therapy because of ongoing gastrointestinal absorption of toxin. Furthermore, it has been hypothesised that administered lipid may even serve to augment enteric absorption through increased plasma affinity for amitriptyline. Contrary to our observed clinical findings, however, in both such scenarios increased plasma amitriptyline would be expected to result in greater manifest toxicity.

Systemic alkalinisation prior to lipid infusion, while resulting in significant contraction in ECG QRS duration, in this case failed to effect improvement in measured haemodynamic performance. Increased pH may, however, have provided the necessary internal milieu for the greatest potential benefit of subsequently administered lipid therapy. Amitriptyline, like bupivacaine, is a pharmacologic weak base (pKa 9.4 [9]) capable of accepting protons to become cationic. Drug ionisation in acidic environments may subsequently result in reduced lipophilicity, precluding maximal potential sequestration to circulating lipid particles. Such a phenomenon has previously been observed by Strichartz et al. who demonstrated increased aqueous:octanol partitioning of bupivacaine with reducing pH [10], and the findings of Mazoit et al. purporting a lowered bupivacaine-lipid binding capacity with acidosis [11]. In the present case, bicarbonate infusion prior to ILE injection likely served to increase the percentage of circulating amitriptyline in the un-ionised state and therefore amenable to lipid sequestration, potentially augmenting the efficacy of the 'lipid sink'. Clearly more study is required to define the role of acid/base status on the potential efficacy of ILE therapy for individual agents.

Animal data exist demonstrating the efficacy of lipid emulsions in rodent and rabbit models of tricyclic antidepressant intoxication [3,4], with early anecdotal human experience apparently supporting utility in desperate clinical circumstances [12,13]. The present case illustrates two potential advantages of ILE utilisation in tricyclic antidepressant cardiotoxicity. Firstly, that ILE resulted in blood pressure elevation subsequent to near normalisation of ECG QRS parameters with bicarbonate therapy suggests benefit beyond that afforded by hypertonic saline solutions alone. Tricyclic antidepressant toxicity is notable for a plethora of disruptions to intracellular function, including dose-dependent myocardial depression in contractile force independent of effects on cardiac conduction [14]. Administered lipid therapy, through direct effects on myocyte high-energy phosphate production [7], increased intracellular calcium concentration [3], and/or indirectly via enhanced myocardial toxin washout, may have contributed significantly to the improvements observed. Recent reports of a lesser tonic, and use-dependent, sodium channel blockade when fatty acids are co-applied with bupivacaine in voltage clamp models in vitro have furthermore suggested direct modulation of cardiac sodium channel function by lipids in local-anaesthetic toxicity [15]. While untested, potential exists for similar modulation to occur in TCA-induced sodium channel blockade.

Secondly, injection of ILE in this case curtailed the requirement for catecholamine-based vasopressors and/or inotropes for the duration of this patient's admission. Avoidance of agents known to be associated with both increased myocardial oxygen consumption and inherent arrhythmogenicity [16] might be considered beneficial in these clinical circumstances.

The clinical features and returned drug levels in this case are consistent with amitriptyline being the prime xenobiotic responsible for manifest toxicity in this case. However, other intoxicants may have contributed to this clinical picture. In particular, some contribution from quinapril, metoprolol, or quetiapine to ongoing hypotension post-sodium bicarbonate administration is possible. Quinapril, however, seems less likely to have responded to lipid emulsion given its low-moderate lipid solubility [5]. Similarly, reversal of metoprolol intoxication seems less likely given both the absence of initial bradycardia and failure of observed response to ILE treatment in experimental models of metoprolol-induced hypotension [17]. Conversely, quetiapine poisoning has been associated with hypotension [18] and is moderately lipophilic with logP of 2.1 [5]. As such, potential exists for ILE therapy to ameliorated quetiapine toxicity in a similar fashion to that proposed for amitriptyline. In the present case, however, we are unable to comment further on the potential contribution to outcomes of this agent because of the failure to perform a quetiapine assay. Literature reports of the response to lipid treatment for isolated quetiapine-induced central nervous system depression alone, however, are variable [19,20].

Given multiple ingested intoxicants and prior administered therapies, it is impossible to definitively attribute the improvements observed in this case to ILE alone. Nevertheless, given the chronology of recovery and presented laboratory metrics, it seems likely that ILE contributed significantly to the favourable outcome observed. Further systematic reporting of individual cases and prospective clinical study is required to determine the role of ILE in human tricyclic antidepressant toxicity.

## Abbreviations

ECG: electrocardiogram; GCS: Glasgow Coma Scale; ILE: intravenous lipid emulsion; TCA: tricyclic antidepressant;

## Consent

Written informed consent was obtained from the patient for publication of this case report and any accompanying images. A copy of the written consent is available for review by the Editor-in-Chief of this journal.

## Competing interests

The authors declare that they have no competing interests.

## Authors' contributions

MH and GC contributed equally to the manuscript generation and revision.

## Authors' information

Dr. Martyn Harvey (MD, FACEM) is an Emergency Physician, Director of Emergency Medicine Research, and Clinical Senior Lecturer (Hon) at Waikato Hospital in Hamilton, New Zealand. Dr. Grant Cave (FACEM, FJFICM) is an Emergency Physician, Intensivist, and Clinical Senior Lecturer (Hon) at Hutt Hospital, Lower Hutt, New Zealand.
